# Prevalence and mechanisms of environmental hyperoxia-induced thermal tolerance in fishes

**DOI:** 10.1098/rspb.2022.0840

**Published:** 2022-08-31

**Authors:** T. J. McArley, D. Morgenroth, L. A. Zena, A. T. Ekström, E. Sandblom

**Affiliations:** Department of Biological and Environmental Sciences, University of Gothenburg, PO Box 463, 405 30 Gothenburg, Sweden

**Keywords:** thermal tolerance, oxygen, hyperoxia, aerobic performance, cardiac function, cardiorespiratory performance

## Abstract

Recent evidence has suggested environmental hyperoxia (O_2_ supersaturation) can boost cardiorespiratory performance in aquatic ectotherms, thereby increasing resilience to extreme heat waves associated with climate change. Here, using rainbow trout (*Oncorhynchus mykiss*) as a model species, we analysed whether improved cardiorespiratory performance can explain the increased thermal tolerance of fish in hyperoxia (200% air saturation). Moreover, we collated available literature data to assess the prevalence and magnitude of hyperoxia-induced thermal tolerance across fish species. During acute warming, O_2_ consumption rate was substantially elevated under hyperoxia relative to normoxia beyond 23°C. This was partly driven by higher cardiac output resulting from improved cardiac contractility. Notably, hyperoxia mitigated the rise in plasma lactate at temperatures approaching upper limits and elevated the critical thermal maximum (+0.87°C). Together, these findings show, at least in rainbow trout, that hyperoxia-induced thermal tolerance results from expanded tissue O_2_ supply capacity driven by enhanced cardiac performance. We show 50% of the fishes so far examined have increased critical thermal limits in hyperoxia (range: 0.4–1.8°C). This finding indicates environmental hyperoxia could improve the ability of a large number of fishes to cope with extreme acute warming, thereby increasing resilience to extreme heat wave events resulting from climate change.

## 1. Introduction

Fish living in shallow aquatic habitats, particularly those that are fully or partially enclosed (e.g. shallow lakes, ponds, streams, estuaries and intertidal rock pools), are regularly exposed to acute warming events. The most extreme of such events occur during heat wave weather conditions and are typically characterized by a daily cycle where water temperature ramps up to a peak over several hours during the day and then cools overnight or on the incoming tide. Although generally considered thermally tolerant, fishes occupying habitats where such acute thermal ramping occurs are under the threat of increasingly frequent and more extreme heatwave events due to climate change [1–3]. Compounding the problem is that behavioural avoidance of high temperatures (e.g. moving to deeper, cooler water) may not be possible in these habitats. An important feature of these environments, however, is that they often support high densities of photosynthetic aquatic plants, algae and seaweeds. Thus, when extreme high temperatures arise (i.e. on hot and sunny days), O_2_ levels are likely to become hyperoxic (supersaturated with O_2_) due to high rates of photosynthesis [4–6]. Indeed, in a recent review, we identified a number of examples of co-occurring acute warming and hyperoxia in shallow aquatic habitats that would support fish populations [7]. Moreover, a thorough assessment of water oxygenation and temperature in shallow Black Sea habitats demonstrated the highest water temperatures frequently co-occur with hyperoxia [8]. Although available research is limited, there is increasing recognition that naturally occurring hyperoxia could provide an ‘ecological refuge’ improving thermal tolerance of fish and other aquatic ectotherms [7–10].

Beyond its ecological relevance, a physiologically based theoretical hypothesis as to why hyperoxia may affect warming tolerance in fish has been proposed. The oxygen and capacity limited thermal tolerance hypothesis places an inability of the cardiorespiratory system to meet tissue O_2_ demand at high temperatures as the central pillar dictating the upper thermal tolerance limits of aquatic ectotherms [11,12]. At critical thermal limits, tissue O_2_ demand is proposed to exceed tissue O_2_ supply, leading to functional tissue hypoxia and time-limited survival dependent on anaerobic metabolism [12,13]. Tissue O_2_ supply is commonly assessed as the rate of O_2_ consumption (ṀO_2_), which, according to the Fick principle, is a product of cardiac output (the rate of blood flow to the tissues) multiplied by the arterial-venous O_2_ content difference (tissue O_2_ extraction from the blood (A-V O_2_ content difference)) [14]. In fish, it is proposed that limitations of maximal cardiac output primarily dictate ceilings of tissue O_2_ supply capacity during warming exposure [15,16]. A prediction stemming from this is that environmental conditions that increase maximal cardiac output during warming will also improve tissue O_2_ supply capacity and may lead to better thermal tolerance. Hyperoxia is one such environmental condition that facilitates improved tissue O_2_ supply (i.e. higher ṀO_2_) at temperatures approaching critical thermal limits in fish [9,17], and there is evidence this is driven by increased cardiac output [18]. While this may explain why hyperoxia improves acute thermal tolerance in some fish [8], a direct link between increased tissue O_2_ supply capacity, higher cardiac output, mitigation of anaerobiosis and improved upper thermal tolerance is yet to be demonstrated.

In the first part of this study, using rainbow trout (*Oncorhynchus mykiss*) as a model species, we aimed to establish the mechanistic basis of a putative improvement of acute upper thermal limits of fish in hyperoxia. We predicted that should thermal tolerance improve with hyperoxia, this would be associated with greater scope to increase ṀO_2_ during thermal ramping and a consequent mitigation of anaerobiosis. While environmental hyperoxia increases arterial O_2_ partial pressure (P*_a_*O_2_) in fish [7], haemoglobin is normally fully saturated in normoxia [19], theoretically leaving little scope for environmental hyperoxia to increase arterial O_2_ content further. Moreover, hyperoxia typically increases venous O_2_ partial pressure (P*_v_*O_2_) [7,18], which would tend to increase venous blood O_2_ content. Thus, in the context of the Fick principle, we predicted that increased ṀO_2_ with environmental hyperoxia would primarily result from increased cardiac output rather than a higher A-V O_2_ content difference. In turn, we predicted that improved cardiac output would be driven by increased cardiac contractility (stroke volume), and that this would be associated with elevated P*_v_*O_2_ and therefore an enhanced cardiac O_2_ supply due to a steeper O_2_ diffusion gradient between the returning venous blood and heart tissue [20]. To assess these predictions, we fitted rainbow trout with a ventral aortic blood flow probe and a venous cannula to allow simultaneous measurements of ṀO_2_, cardiac function, venous blood oxygenation and blood parameters (lactate and haematology) during thermal ramping to the critical thermal maximum (CT_max_) under hyperoxia (200% air saturation) or normoxia.

Hyperoxia does not always increase the thermal tolerance of fish examined under controlled laboratory conditions [9,17]. Thus, it is unclear whether a recently proposed idea that natural environmental hyperoxia can enhance the resilience of aquatic ectotherms to more extreme acute warming [8] is relevant in a broad range of fishes and environments. In the second part of this study, we therefore synthesized the existing literature concerning the impact of hyperoxia on acute upper thermal tolerance limits in fish. In doing so, we aimed to assess: (i) whether improved thermal tolerance in hyperoxia is a general response observed in a broad range of fishes and (ii) the magnitude of environmental hyperoxia-induced increases in thermal tolerance in fishes.

## 2. Methods

### (a) Experimental animals and holding conditions

The rainbow trout (mean body mass of 896.5 ± 47.5 g and 901.8 ± 73.9 g at the time of experimentation in the normoxia and hyperoxia treatments, respectively) used in this study were of mixed sex and obtained from a commercial trout farm (Vänneåns Fiskodling AB, Halland, Sweden). Prior to experimentation, they were held in two 400 l tanks supplied with recirculated freshwater (air saturated, approx. 10°C and 12 : 12 h light cycle) for a period of at least four weeks of laboratory acclimation. They were fed commercial aquaculture feed (7 mm, Protec Trout pellets, Skretting, Norway) twice a week, but food was withheld for a period of 3 days prior to experimentation.

### (b) Surgery and instrumentation

To measure cardiac output, heart rate and stroke volume, a 2.5 mm Transonic transit-time blood flow probe (L type; Transonic Systems, Ithaca, NY) was placed around the ventral aorta to allow recordings of blood flow. Anaesthesia and surgical methods for fitting the flow probe were identical to McArley *et al*. [21]. The ducts of Cuvier were then cannulated with a PE50 catheter to allow venous blood sampling as previously described by Sandblom *et al*. [22].

### (c) Experimental protocol prior to thermal ramping

Following surgery, individual fish were placed into respirometers held in 120 l aquariums receiving a constant flow of approximately 10°C recirculated freshwater from the main holding tank supply. After the fish was placed in the respirometer, an O_2_ level of approximately 200% air saturation was established for the hyperoxic treatment by bubbling water with O_2_, while the normoxia treatment (approx. 100% air saturation) was maintained by bubbling air. These O_2_ treatment conditions were then maintained for the remainder of the protocol. Fish recovered from surgery for approximately 22 h, at which point they were removed from the respirometers and exhaustively exercised under normoxia or hyperoxia by manual chasing for a period of 5 min. They were then returned to the respirometers and allowed to recover for 21 h prior to the onset of thermal ramping. During the time prior to thermal ramping, five approximately 250 µl venous blood samples (approx. 1.25 ml total and approx. 3.3% of total blood volume) had been drawn from which the physiological parameters measured are not reported in the current study; the data pertaining to these samples are reported in McArley *et al*. [21]. The blood sample drawn (sixth sample) and the ṀO_2_ and cardiac function measured at the end of the post-exhaustive exercise recovery period are used as routine values at 10°C in the current study (see electronic supplementary material, figure S1 for a visual outline of the experimental protocol). At this point, following exhaustive exercise, ṀO_2_, cardiac output and all blood parameters measured had recovered to pre-exhaustive resting levels under both normoxia and hyperoxia [21]. Thus, although previously exposed to exhaustive exercise, fish in both O_2_ treatments were in a similarly well-rested and recovered state prior to thermal ramping. This allowed us to reduce the total number of research animals used, while maximizing the data collected, in accordance with 3R principles.

### (d) Thermal ramping protocol

Thermal ramping involved step-wise increases in temperature, which were achieved by heating the water supply to the respirometers housing fish with a water heater controlled with a thermostat. The mean water O_2_ level inside the respirometer throughout the entire thermal ramping protocol was 96.9 ± 0.2% air saturation and 208.8 ± 2% air saturation in the normoxia and hyperoxia treatment, respectively. These are referred as normoxia (approx. 100% air saturation) and hyperoxia (approx. 200% air saturation) for the remainder of this paper. Initially, temperature was increased from 10°C to 15°C and then 15°C to 20°C at a rate of 5°C h^−1^. At 15°C and 20°C, ṀO_2_ and cardiac variables were measured for approximately 20 min once temperature stabilized (e.g. temperature was increased from 10°C to 15°C in 40 min; then measurements were taken at 15°C for 20 min). A venous blood sample (approx. 250 µl) was also drawn at the end of the 20°C measurement period. From 20°C, the rate of heating was reduced to 2°C h^−1^. ṀO_2_ and cardiac variables were measured for a period of approximately 20 min at 22°C and then for 20 min with every 1°C increase in temperature beyond 22°C. A venous blood sample was also taken at the end of the 24°C and 26°C temperature steps. Thermal ramping continued until the loss of equilibrium (i.e. an inability to maintain a stable, upright body position) occurred for a period of 10 s, which was defined as the critical thermal maximum (CT_max_) [23]. A final blood sample was taken at CT_max_ prior to the fish being removed from the respirometer and euthanized with a concussive blow to the head.

### (e) Respirometry for ṀO_2_ measurement and data acquisition for cardiac variables

ṀO_2_ was measured using intermittent stop-flow respirometry [24]. Respirometer design, respirometry data acquisition equipment and software, and calculation of ṀO_2_ were identical to McArley *et al*. [21]. Briefly, in the ‘closed’ measurement phase, the linear decline in water O_2_ level (sampled at 10 Hz with a fibre optic probe) within a sealed 10 l PVC respirometer was used to calculate ṀO_2_ at each thermal ramping temperature step. The *R*^2^ for the slope of the linear decline in water O_2_ level within the respirometer was greater than 0.98 for the majority of measurement cycles and never below 0.95. Three ‘closed’ phase measurement cycles (2–5 min) interspersed with a ‘flushing’ period (5–8 min) were run at each temperature. Background O_2_ consumption was assessed at 10°C at the start of the protocol and at the temperature of CT_max_. At 10°C, a positive background slope, which likely related to a small increase in temperature (approx. 0.15°C) within the sealed respirometer during ‘closed’ measurement cycles, was detected. The source of this heat was almost certainly the mixing pump connected to the respirometer. This positive slope, however, was reduced in a linear fashion as temperature increased during thermal ramping, and it often became slightly negative at the highest temperatures. Thus, to estimate background O_2_ consumption, a linear regression between temperature and the background slope measured at the start (10°C) and end (CT_max_ temperature) of the protocol was used to calculate the background slope at each thermal ramping temperature. These slopes were then added (positive slope) or subtracted (negative slope) from the measurement cycle slopes used to calculate ṀO_2_.

The signal from the Transonic blood flow probe was sampled at 10 Hz using identical equipment and software as McArley *et al*. [21], and the probe was bench calibrated between temperatures of 10°C to 26°C according to the manufacturer's instructions (see Morgenroth *et al*. [25] for a detailed description of the calibration set-up). The flow probe signal was recorded continuously throughout thermal ramping, but only data pertaining to periods of ṀO_2_ measurement, once temperature had stabilized at each thermal ramping step, was used to assess cardiac parameters.

### (f) Calculation of cardiorespiratory variables

Cardiac output was determined from blood flow data and normalized to body mass (ml min^−1^ kg^−1^), and heart rate was determined from the pulsatile blood flow measurements. Cardiac stroke volume (ml heart beat^−1^) was calculated by dividing cardiac output by heart rate. Routine and maximal values for cardiorespiratory variables are reported in this study. Routine values for ṀO_2_ (ṀO_2−ROU_) are the mean of three measurements taken at each thermal ramping temperature step. For cardiac variables, routine values are determined from the mean of three sections of flow trace recorded at the same time as ṀO_2-ROU_ (i.e. routine cardiac variables are tied to ṀO_2-ROU_). Maximal ṀO_2-ROU_ during thermal ramping was taken as the highest ṀO_2-ROU_ (mean of three ṀO_2_ values) recorded at any temperature. In all fish, this occurred at temperatures of 24°C or higher. Like routine cardiac variables, maximal cardiac variables were tied to ṀO_2,_ such that the maximum values for cardiac output, heart rate and stroke volume reported pertain to the same time when maximal ṀO_2-ROU_ was recorded. Cardiac variables were tied to ṀO_2_ because a main focus of the experiment was to determine whether predicted differences in ṀO_2_ between normoxia and hyperoxia were driven by differences in cardiac function. Using the tied ṀO_2_ and cardiac output measurements, routine and maximal A-V O_2_ content difference was estimated by rearrangement of the Fick equation:

2.1 $${\text{A-V O}}_{2}\;\mathrm{content}\;\mathrm{difference}=\frac{\dot{M}{\text{O}}_{2}}{\mathrm{cardiac}\;\mathrm{output}}.$$

### (g) Blood analysis

Venous blood samples (approx. 250 µl) were drawn prior to thermal ramping at 10°C and during thermal ramping at 20°C, 24°C and 26°C. In each sample, P*_v_*O_2_, haemoglobin concentration ([Hb]), haematocrit (Hct) and plasma lactate were assessed using identical equipment and protocols to McArley *et al*. [21].

### (h) Statistics

**Figure 1.**
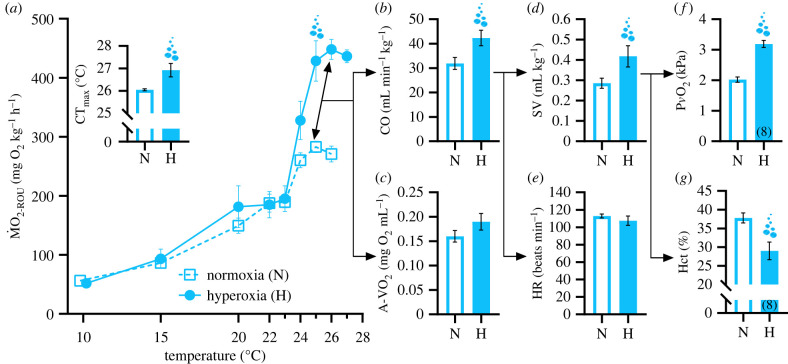
Thermal tolerance and cardiorespiratory performance of rainbow trout (*Oncorhynchus mykiss*) facing acute warming under hyperoxia (200% air saturation) or normoxia. All values are means ± s.e.m. (*n* = 9 unless indicated by bracketed numbers). (*a*) Routine mass-specific O_2_ consumption rate (ṀO_2-ROU_), with critical thermal maximum (CT_max_: the temperature at which fish could no longer maintain a stable, upright body orientation) shown in the insert. The bubbles in (*a*) indicate a significant difference (*p* < 0.05) in ṀO_2-ROU_ at 25°C as assessed by mixed two-way ANOVA (see electronic supplementary material, figure S1 for statistical results); (*b*–*e*) show variables at maximal ṀO_2-ROU,_ which occurred at 24.9 ± 0.26 and 25.7 ± 0.24°C in normoxia and hyperoxia, respectively. (*b*) Cardiac output (CO); (*c*) arterial-venous O_2_ content difference (A-V O_2_) estimated by the Fick equation; (*d*) cardiac stroke volume (SV) and (*e*) heart rate (HR); (*f*,*g*) show venous O_2_ partial pressure (P*v*O_2_) and haematocrit (Hct) in blood samples drawn via a cannula at 26°C. For the normoxia treatment, seven out of nine blood samples for the 26°C comparison were drawn immediately upon reaching CT_max_. In the hyperoxia treatment, the eight fish included in the 26°C comparison did not reach CT_max_ for the entire temperature step. The sample size of 8 at 26°C reflects the fact that one fish in hyperoxia reached CT_max_ at 25°C. Bubbles on plots (*b–g*) indicate a significant difference (*p* < 0.05) as assessed by independent-samples *t*-tests, with the exception of CT_max_ and heart rate for which comparisons were made using a Mann–Whitney U test (see electronic supplementary material, table S1 for statistical results). (Online version in colour.)

**Figure 2.**
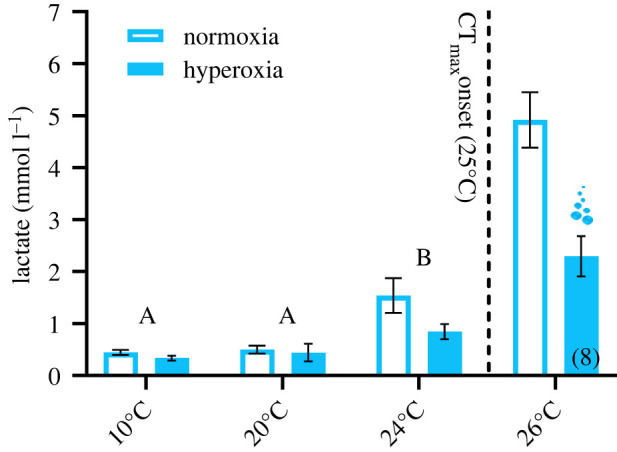
Plasma lactate concentration in rainbow trout (*Oncorhynchus mykiss*) facing acute warming under hyperoxia (200% air saturation) or normoxia. All values are means ± s.e.m. (*n* = 9 unless indicated by bracketed numbers). Over the 10–24°C range, letters represent significant differences (*p* < 0.05) between temperatures across O_2_ treatments as assessed by mixed two-way ANOVA (temperature: *F*_(1.53, 24.53)_ = 30.95, *p* < 0.001). At 26°C, bubbles represent a significant difference (*p* < 0.05) between O_2_ levels as assessed by an independent-sample *t*-test (see electronic supplementary material, table S1 for statistical results). For the normoxia treatment, seven out of nine blood samples for the 26°C comparison were drawn immediately upon reaching the critical thermal maximum (CT_max_: the temperature at which fish could no longer maintain a stable, upright body orientation). In the hyperoxia treatment, the eight fish included in the 26°C comparison did not reach CT_max_ for the entire temperature step. The sample size of 8 at 26°C reflects the fact that one fish in hyperoxia reached CT_max_ at 25°C. (Online version in colour.)

All analyses were performed using GraphPad Prism (version 9.10), with statistical significance accepted at *p* < 0.05. For repeated measures analyses, a violation of sphericity was assumed and Geisser–Greenhouse adjusted *p*-values and *F*-tests are reported. Cardiorespiratory variables (ṀO_2-ROU,_ cardiac output, heart rate, stroke volume and A-V O_2_ content difference) were analysed in two ways. First, routine responses were compared between normoxia and hyperoxia up to a temperature of 25°C (i.e*.* prior to any fish reaching CT_max_) using mixed two-way analysis of variance (ANOVA). For cardiac variables and A-V O_2_ content difference, the findings of these analyses are presented in electronic supplementary material, figure S2. ṀO_2-ROU_, cardiac output, stroke volume and A-V O_2_ content difference were natural log transformed to ensure homoscedasticity and normality of residuals, with the exception of ṀO_2-ROU_ (figure 1*a*) where the analysis was performed on transformed values despite left skew remaining. In the second analysis of cardiorespiratory variables, maximal ṀO_2-ROU_ (natural log transformed), cardiac output, stroke volume (natural log transformed) and A-V O_2_ content difference (natural log transformed) at maximal ṀO_2-ROU_, were compared between O_2_ treatments using independent-samples *t*-tests (figure 1*b*–*e*). For heart rate at maximal ṀO_2-ROU_, medians were compared using a Mann–Whitney *U*-test. Blood variables (PvO_2,_ Hct, [Hb] and plasma lactate) were also assessed using two analyses. First, at temperatures that all fish reached prior to CT_max_ (10°C, 20°C and 24°C), comparisons were made between O_2_ treatments using mixed two-way ANOVA. For [Hb] and Hct, the analysis was run despite non-normality among residuals, as this could not be eliminated through data transformation. The two-way ANOVAs for P*_v_*O_2_ and plasma lactate were performed following a natural log transformation to correct for homoscedasticity or non-normality among residuals. The findings of these analyses, with the exception of plasma lactate (figure 2), are presented in the electronic supplementary material, figure S3. In the second analysis of blood variables, independent-samples *t*-tests were used to compare blood samples drawn at 26°C. In the normoxic treatment, this included values from two fish at 26°C prior to CT_max_ and values from seven fish that reached CT_max_ at 26°C (i.e. blood was sampled immediately following the loss of equilibrium in seven fish at 26°C). For the hyperoxic treatment, values at 26°C represented eight fish that did not reach CT_max_ for the entire duration of the 26°C temperature step (note, one fish in hyperoxia reached CT_max_ at 25°C and therefore was not included in the 26°C comparison). Finally, due to non-normality, median CT_max_ was compared between O_2_ treatments using a Mann–Whitney *U*-test.

## 3. Results and discussion

### (a) Hyperoxia increases thermal tolerance through boosting maximal tissue O_2_ supply capacity

The upper acute thermal limit (CT_max_) was measured to determine if rainbow trout gain a thermal tolerance advantage of hyperoxic (200% air saturation) water oxygenation. In the case of improved CT_max_ with hyperoxia, we predicted a corresponding increase in tissue O_2_ supply capacity. To assess this prediction, ṀO_2-ROU_ (an estimate of whole-animal tissue O_2_ supply capacity) was measured in normoxia and hyperoxia during acute thermal ramping (approx. 2°C h^−1^) to the temperature at which fish could no longer maintain equilibrium (i.e. a stable, upright body orientation; CT_max_). In hyperoxia, the CT_max_ of rainbow trout was significantly higher than in normoxia (figure 1*a*). As predicted, the elevation of CT_max_ with hyperoxia was associated with a greater tissue O_2_ supply capacity. Indeed, there was a striking difference in the ability of normoxia and hyperoxia-exposed rainbow trout to increase ṀO_2-ROU_ beyond 23°C (figure 1*a*). This was reflected by hyperoxia-treated fish having a 52% higher ṀO_2-ROU_ at 25°C (figure 1*a*). Moreover, the maximal ṀO_2-ROU_ during thermal ramping, which was observed at 24.9 ± 0.26 and 25.7 ± 0.24°C in normoxia and hyperoxia, respectively, was 58% higher in hyperoxia (292.8 ± 6.6 mg O_2_ kg^−1^ h^−1^ in normoxia versus 462.1 ± 21.6 mg O_2_ kg^−1^ h^−1^ in hyperoxia; *p* < 0.001, see electronic supplementary material, table S1 for statistical results). It is proposed that acute warming limits in aquatic ectotherms are set by temperature-dependent performance ceilings of maximum tissue O_2_ supply capacity, and that when this ceiling is reached, survival becomes time limited and increasingly reliant on unsustainable anaerobic ATP production [11,12]. Here, we show that the substantial benefit of hyperoxia to tissue O_2_ supply capacity mitigated anaerobiosis at high temperatures. Indeed, at 26°C, a temperature where seven out of nine fish in normoxia but only one of nine fish in hyperoxia had reached CT_max_, plasma lactate levels—a by-product of anaerobic metabolism—were significantly lower in hyperoxia than normoxia (figure 2). This finding indicates that higher CT_max_ in hyperoxia may have resulted from improved O_2_ supply capacity shifting the point at which anaerobic ATP production became unsustainable to a higher temperature. As CT_max_ is marked by a loss of coordination, which likely involves some form of neural impairment, severe anaerobiosis in brain tissue may be a candidate for the proximate cause of loss of equilibrium at high temperatures. In support of this, unsustainable anaerobic respiration (i.e. exhaustion of ATP, depletion of glycogen and marked increases in lactate) in brain tissue was identified as a key characteristic at the loss of equilibrium in hypoxia exposed sculpin species [26].

### (b) Hyperoxia boosts heart blood pumping capacity

Limits on the maximum blood pumping capacity of the heart have been proposed as the primary determinant of ceilings in tissue O_2_ supply during warming exposure in fish [16]. Moreover, as haemoglobin is normally fully saturated in normoxia [19], hyperoxia is unlikely to increase arterial O_2_ content and therefore should not influence A-V O_2_ content difference [27]. Thus, we predicted that any expansion of tissue O_2_ supply capacity (i.e. higher ṀO_2_) in hyperoxia during thermal ramping would be driven by higher cardiac output. Confirming this prediction, higher ṀO_2-ROU_ at 25°C in hyperoxia occurred alongside a 29% elevation of cardiac output at the same temperature (*p* < 0.05; electronic supplementary material, figure S2). This also occurred at maximal ṀO_2-ROU,_ where cardiac output was 33% higher with hyperoxia (*p* < 0.05; figure 1*b*). Because heart rate was similar between treatment groups across temperatures (electronic supplementary material, figure S1) and at maximal ṀO_2-ROU_ (figure 1*e*), the higher cardiac output with hyperoxia likely reflected an increased cardiac contractility. Indeed, a strong trend for higher stroke volume with hyperoxia existed at temperatures beyond 24°C (electronic supplementary material, figure S2), and stroke volume was 46% higher with hyperoxia at maximal ṀO_2-ROU_ (*p* < 0.05; figure 1*d*). Increased cardiac contractility with hyperoxia may have been the result of an improved cardiac O_2_ supply. The partial pressure of O_2_ in venous blood was elevated in hyperoxia across thermal ramping temperatures (electronic supplementary material, figure S3) and was approximately 1.2 kPa higher at 26°C (figure 1*f*). Although the difference in P*_v_*O_2_ between normoxia and hyperoxia was relatively small, it existed in a range (normoxia approx. 2 kPa and hyperoxia approx. 3.2 kPa; figure 1*f*) below the threshold (approx. 6 kPa) where progressive declines in maximal cardiac output with falling perfusate O_2_ partial pressure begin in rainbow trout perfused heart preparations [28]. Thus, as P*_v_*O_2_ was below the threshold known to impair maximal cardiac output *in situ*, it is plausible that the higher *in vivo* P*_v_*O_2_ in hyperoxia could have contributed to improved cardiac performance by steepening the O_2_ diffusion between blood entering the heart lumen and the spongy myocardium. Elevated P*_v_*O_2_ in hyperoxia was also observed alongside improved stroke volume during thermal ramping in European perch (*Perca fluvialitis)* [18].

The interpretation made here that enhanced cardiac contractility with hyperoxia is driven by higher P*_v_*O_2_ is complicated by two factors. First, rainbow trout also have a coronary circulation, which supplies oxygenated arterial blood directly from the gills to the outer compact myocardium [29]. We have recently observed that arterial O_2_ partial pressure (P*_a_*O_2_) is approximately 16 kPa higher under hyperoxia (200% air saturation) relative to normoxia following exhaustive exercise in rainbow trout (T.J.M. 2022, unpublished data). Moreover, elevated P*_a_*O_2_ is a common response to hyperoxia in almost all fish so far examined [7]. Thus, it is likely that hyperoxia also results in increased P*_a_*O_2_ during thermal ramping and that improved contractility may be due to a steepened O_2_ diffusion gradient between the coronary blood and the compact myocardium of the heart. The second complicating factor is that hyperoxia also reduced Hct in the current study (from 32% to 25% at 24°C, and from 37% to 29% at 26°C; electronic supplementary material, figure S2; figure 1*g*). In European seabass (*Dicentrarchus labrax*), experimental anaemia that reduced Hct from 42% to 20% increased peak cardiac output during thermal ramping by 42% [30]. Thus, some of the approximately 33% increase in peak cardiac output observed here with hyperoxia may have been the result of the lower Hct rather than being solely related to improved cardiac O_2_ supply directly influencing contractility. In hyperoxia, due to the mitigating influence of increased blood PO_2,_ it may be that an active reduction in Hct can take place without compromising aerobic performance. The potential benefit of this is that lower Hct reduces blood viscosity and could therefore lower the energetic costs of the heart [31].

A somewhat perplexing finding of our study is that, despite lower [Hb] and higher P*_v_*O_2_ (electronic supplementary material, figure S3), there was a trend for a 19% higher A-V O_2_ content difference (Fick estimated) with hyperoxia at maximal ṀO_2-ROU_ (figure 1*c*)_._ If haemoglobin were fully saturated in arterial blood under both O_2_ levels, this finding would be inexplicable. Our working hypothesis is that this is not necessarily the case. In rainbow trout exposed to thermal ramping under normoxia, it is known that haemoglobin O_2_ saturation can fall to approximately 75% at high temperatures [32]. Moreover, although haemoglobin O_2_ saturation was unaffected, P*_a_*O_2_ fell from approximately 18.6 kPa to approximately 9.5 kPa in heat shocked (13°C to 25°C in 4 h) rainbow trout [33]. As noted earlier, we now know that hyperoxia drastically increases P*_a_*O_2_ relative to normoxia following exhaustive exercise in rainbow trout (T.J.M. 2022, unpublished data). If this also occurs during thermal ramping, it may afford protection against collapsing haemoglobin O_2_ saturation and arterial O_2_ content as CT_max_ is approached. This hypothesis remains speculative, however, and follow-up studies measuring arterial oxygenation under normoxia and hyperoxia during thermal ramping are required.

### (c) Prevalence and magnitude of hyperoxia-induced thermal tolerance in fishes

Recent evidence, as was the case in the current study, has shown hyperoxia can increase the critical upper thermal limits of fish inhabiting shallow, tropical coastal environments, suggesting photosynthetically driven O_2_ supersaturation could increase the resilience of fishes living in such habitats to more extreme heat waves associated with climate change [8]. In our own past work, however, hyperoxia has failed to influence upper critical thermal limits [9,17], indicating hyperoxia-induced thermal tolerance may be species and context specific. To understand the generality of the phenomenon of hyperoxia-induced thermal tolerance, we collated existing literature data from studies that examined the influence of hyperoxia on upper critical limits in fish. Ten publications (present study included) were identified (table 1). These studies included 20 species ranging from exclusively tropical to Antarctic climatic regions. Of the 20 species examined, a significant elevation of CT_max_ with hyperoxia has been demonstrated in nine species (table 1). In one further species, hyperoxia appeared to increase upper thermal tolerance (*Carrisius auratus* +1°C) but no statistical comparison could be made due to experimental design (table 1). The magnitude of improvement in thermal tolerance with hyperoxia ranged from +0.4°C to +1.8°C (table 1). This finding indicates naturally occurring hyperoxia could benefit thermal tolerance in a large number of fishes and potentially improve resilience to more extreme heat wave events due to climate change. A caveat of this conclusion, however, is that almost all studies have failed to replicate naturally occurring hyperoxic episodes. The reason for this is that most have been performed in a purely mechanistic rather than ecological context, where replicating naturally occurring O_2_ levels and heating rates have not been a priority. The best effort so far has been that of Giomi *et al*. [8] who matched experimental O_2_ levels and heating rates to extensive monitoring data from relevant ecosystems. These authors' study demonstrates the largest benefits of environmental hyperoxia to critical thermal limits among the available literature.

**Table 1.** The effect of environmental hyperoxia on upper thermal tolerance limits in fish. CT_max_ difference = CT_max_ in hyperoxia - CT_max_ in normoxia (a positive number shows higher CT_max_ in hyperoxia), na = no statistical comparison available, ns = not stated. Note: Giomi *et al*. [8] reported the temperature at which 50% of fish became unresponsive (LT50) as a measure of thermal tolerance. As LT50 was determined by sigmoidal regression, the separation of 95% confidence intervals between the regressions in normoxia and hyperoxia was taken as a statistically significant difference. All other studies reported CT_max_ (i.e. the temperature of loss of equilibrium). Ecotype: FW = freshwater, M = marine, BW = brackish water. Climatic region was determined from the latitudinal distribution listed for each species on FishBase (https://www.fishbase.de/).

species	ecotype	climatic region	acclimation temperature (°C)	heating rate (°C h^−1^)	O_2_ level (% air saturation)	CT_max_ difference (°C)	*p* < 0.05	reference
*Carassius auratus*	benthopelagic; FW/BW	subtropical-temperate	17	109	200	+0.9	na^a^	[13]
*C. auratus*			17	109	450	+1	na	
*C. auratus*			27	73	200	+0.21	na	
*C. auratus*			27	73	450	+0.81	na	
*Fundulus notatus*	benthopelagic, FW	subtropical-temperate	30	20	160	−0.01	no	[34]
*Notropis lutrensis*	benthopelagic, FW	subtropical-temperate	30	20	160	−0.53	no	
*Pimephales vigilax*	benthopelagic, FW	subtropical-temperate	30	20	160	−0.16	no	
*Fundulus heteroclitus*	benthopelagic, FW/M/BW	subtropical-temperate	15	18	ns	+0.3	no	[35]
*Perca fluviatilis*	benthopelagic (Biotest population), FW/BW	temperate	23	2	200	+0.6	no	[17]
*P.fluviatilis*	benthopelagic, FW/BW	temperate	17	2	200	+1.1	yes	[18]
*Chaenocephalus aceratus*	benthopelagic, M	Antarctic	0.5	4	240	+0.12	no	[36]
*Notothenia coriiceps*	benthopelagic, M	Antarctic	0.5	4	240	+0.74	no	
*Bellapiscis medius*	benthic (intertidal), M	temperate	21	2	200	+0.13	no	[9]
*Forsterygion lapillum*	benthopelagic (intertidal/subtidal), M	temperate	21	2	200	+0.43	yes	
*Atherinomorous* sp.	pelagic, M	tropical	20	2	140	+1.4	yes	[8]
*Dascyllus* sp.	reef associated, M	tropical	20	2	140	+1.8	yes	
*Apistogramma borellii*	benthopelagic, FW	tropical-subtropical	31	12	200	+0.66	no	[37]
*Brycon amazonicus*	benthopelagic, FW	tropical	31	12	200	+1.4	yes	
*Carnegiella strigata*	pelagic, FW	tropical	31	12	200	+0.51	yes	
*Colossoma macropomum*	benthopelagic, FW	tropical	31	12	200	+0.08	no	
*Corydoras pulcher*	benthopelagic, FW	tropical	31	12	200	+0.81	no	
*Corydoras schwartzi*	benthopelagic, FW	tropical	31	12	200	+0.48	yes	
*Paracheirodon axelrodi*	pelagic, FW	tropical	31	12	200	+0.41	yes	
*Oncorhynchus mykiss*	benthopelagic, FW/M/BW	temperate	10	2	200	+0.87	yes	current study

^a^Weatherley [13] did not test statistical significance when comparing CT_max_ but did show a significant increase in the time fish survived exposure to 40°C under hyperoxia relative to normoxia.

In addition to improving upper critical thermal limits in fish, other benefits of environmental hyperoxia may exist at elevated but sub-lethal temperatures. In the current study, the large increase in maximal ṀO_2-ROU_ at elevated temperatures with hyperoxia, probably means that hyperoxia also increases aerobic scope (the difference between resting ṀO_2_ and maximal ṀO_2_) at elevated temperatures. The same expansion of ṀO_2_ with hyperoxia at acutely elevated temperatures has also been observed in European perch and two triplefin fishes [9,17]. Aerobic scope is proposed to represent the metabolic performance window within which fish can perform aerobically demanding activities [38]. The basic principle is that constraint or expansion of aerobic scope by a given environmental factor corresponds to a constraint or expansion of the capacity of an organism to perform aerobically demanding activities such as swimming, feeding, digestion, growth and reproduction [39]. In this context, we propose the apparent expansion of aerobic scope in fish facing acute warming exposure under hyperoxia may represent a sub-lethal metabolic refuge that mitigates severe constraints on aerobic performance that would otherwise occur with acute warming under normoxia. A possible trade-off to the proposed benefit of hyperoxia to aerobic performance, however, could be increased levels of oxidative stress. Indeed, it is known that hyperoxia can increase O_2_ free radical production and cause oxidative damage to tissues in fish (see [7] for a detailed review of this topic). Future studies assessing the benefits of hyperoxia to aerobic performance and thermal tolerance should also consider whether co-occurring hyperoxia and acute warming also impose harmful oxidative stress.

### (d) Conclusions

Recent evidence has demonstrated that naturally occurring environmental hyperoxia can improve upper critical thermal limits in fish and therefore may increase resilience of fish living in heat-vulnerable habitats (e.g. rock pools, shallow estuaries and shallow lakes and ponds) to more extreme acute warming events occurring with climate change. Here, we show hyperoxia also increases CT_max_ in rainbow trout. We demonstrate hyperoxia substantially increases maximal tissue O_2_ supply capacity at elevated temperatures approaching upper critical limits and mitigates anaerobiosis. Moreover, the blood pumping capacity of the heart is boosted with hyperoxia as evidenced by increased cardiac output and stroke volume. Together these findings indicate that hyperoxia can benefit acute thermal tolerance in fish through expanding cardiorespiratory performance and improving tissue O_2_ supply capacity. Our literature review found that environmental hyperoxia increases upper critical thermal limits in half of the fishes so far examined. Thus, naturally occurring environmental hyperoxia could improve upper acute thermal tolerance limits and increase resilience to extreme heat wave events resulting from climate change in a large number of fishes.

## Data Availability

Original data relating to this manuscript is available as electronic supplementary material [40]
